# Self-management support and eHealth for patients and informal caregivers confronted with advanced cancer: an online focus group study among nurses

**DOI:** 10.1186/s12904-017-0238-4

**Published:** 2017-11-21

**Authors:** Vina N. Slev, H. Roeline W. Pasman, Corien M. Eeltink, Cornelia F. van Uden-Kraan, Irma M. Verdonck-de Leeuw, Anneke L. Francke

**Affiliations:** 1 Department of Public and Occupational Health, VU University Medical Center/Amsterdam Public Health research institute, Van Der Boechorststraat 7, 1081 BT Amsterdam, Netherlands; 2 Expertise Center for Palliative Care, Van der Boechorststraat 7, 1081 BT Amsterdam, Netherlands; 3 Department of Hematology, VU University Medical Center, De Boelelaan 1118, 1081 HZ Amsterdam, Netherlands; 4 Department of Clinical Psychology, VU University, Van der Boechorststraat 1, 1081 BT Amsterdam, Netherlands; 5 Department of Otolaryngology - Head & Neck Surgery, VU University Medical Center, De Boelelaan 1118, 1081 HZ Amsterdam, Netherlands; 6 Cancer Center Amsterdam (CCA), De Boelelaan 1118, 1081 HZ Amsterdam, Netherlands; 7 NIVEL, Netherlands Institute for Health Services Research, Otterstraat 118 – 124, 3513 CR Utrecht, Netherlands

**Keywords:** Nurses, Advanced cancer, Self-management support, eHealth

## Abstract

**Background:**

Self-management by patients and informal caregivers confronted with advanced cancer is not self-evident. Therefore they might need self-management support from nurses. This article reports on nurses’ perspectives on self-management support for people confronted with advanced cancer, and nurses’ experiences with eHealth in this context.

**Methods:**

Six online focus groups were organized, with a total of 45 Dutch nurses with different educational levels and working in different care settings. Nurses were asked how they support patients and informal caregivers facing advanced cancer in managing physical and psychosocial problems in their daily life. Questions were also asked regarding the nurses’ experiences with eHealth. Transcripts of the online focus group discussions were analyzed qualitatively following the principles of thematic analysis. The main themes derived from the analyses were ordered according to the elements in the 5 A’s Behavior Change Model.

**Results:**

Within the scope of self-management support, nurses reported that they discuss the background, personal situation, wishes, and needs of advanced cancer patients (‘Assess’ in the 5 A’s model), and they provide information about cancer and specifically the advanced type (‘Advise’). However, nurses hardly give any advice on how patients can manage physical and psychological problems themselves and/or pay any attention to collaborative goal-setting (‘Agree’). Neither do they explain how follow-up can be arranged (‘Arrange’). In addition, they do not appear to pay much attention to self-management support for informal caregivers.

Nurses’ attitudes towards eHealth within the scope of self-management support are positive. They see many advantages, such as allowing advanced cancer patients to stay in charge of their own care and lives. However, nurses also explicitly stressed that eHealth can never be a substitute for personal contact between nurses and patients.

**Conclusions:**

Nurses value self-management support and eHealth for advanced cancer patients and their informal caregivers. However, they seem to disregard important elements in the support of self-management, such as providing practical advice, collaborative goal-setting, and arrangement of follow-up. We recommend further promoting and clarifying the essence and importance of self-management support, including self-management support for informal caregivers.

## Background

Self-management by patients and informal caregivers confronted with a life limiting illness, such as advanced cancer, is not self-evident. Patients might have limited self-management skills, among more, because of their physical deterioration [1]. Alongside physical symptoms and problems, such as pain, fatigue and loss of appetite, patients also have to deal with psychological problems like anxiety and depressive moods. Symptoms and problems which may be severe and progressive over time in patients with an advanced form of cancer [2]. Besides, research literature suggests that incurably ill patients often lack fundamental knowledge and understanding of the progression of their illness, and have limited insight into care opportunities. Aspects which are important for self-management [3]. Patients may therefore require self-management support from healthcare professionals, such as nursing staff.

Informal caregivers who care for patients may also suffer from problems such as depressed moods, anxiety, and/or a decrease in social activities related to their often high care burden [4].

Hence, both patients and informal caregivers may need self-management support. In this study we use Wagner et al.’s definition of self-management support: “[…] Acknowledging the patients' central role in their care, one that fosters a sense of responsibility for their own health. It includes the use of proven programs that provide basic information, emotional support, and strategies for living with chronic illness. […] Using a collaborative approach, providers and patients work together to define problems, set priorities, establish goals, create treatment plans and solve problems along the way.” [5].

Different models have been developed for self-management and self-management support (e.g. Battersby [6], Battersby et al. [7], Lorig et al. [8, 9]). A widely accepted model is the 5 A’s Behavior Change Model, originally developed by the U.S. Department of Health [10], further developed by Glasgow et al. [11], and a point of departure for the Dutch national care standard on self-management [12], as well as for other recent research on self-management and self-management support [13].

**Fig. 1:**
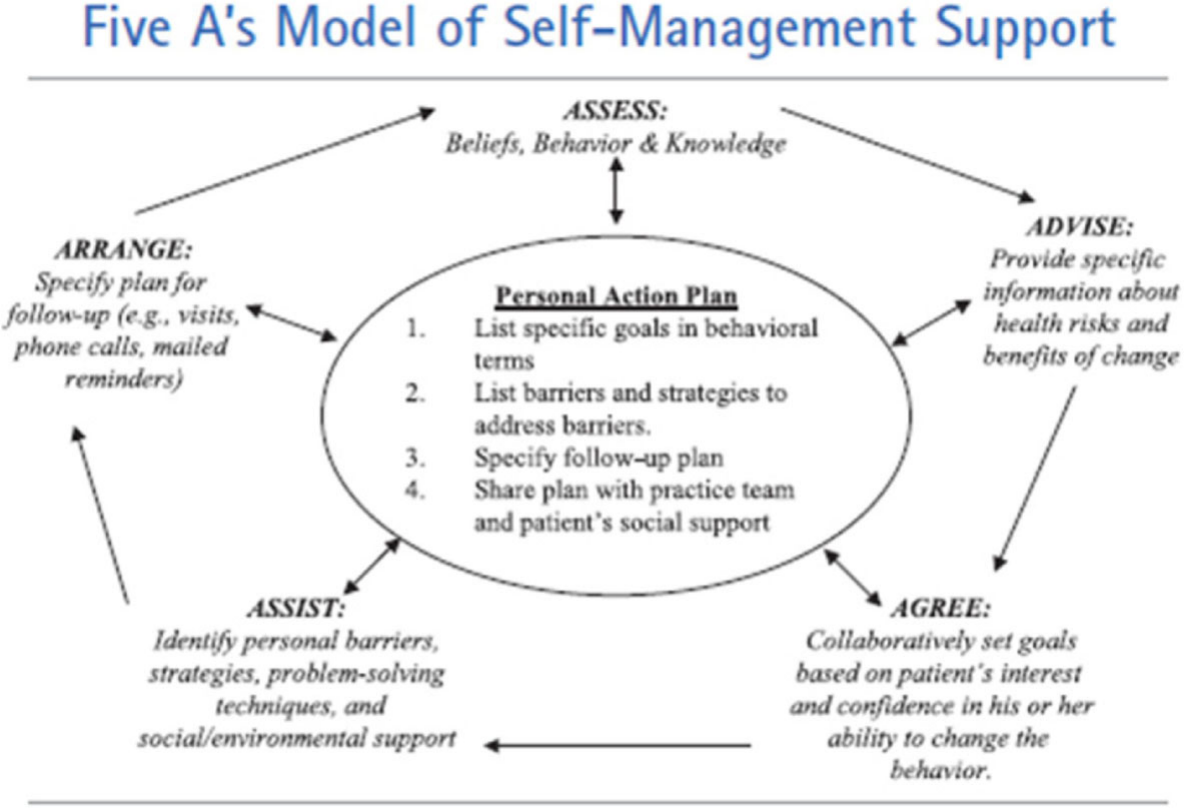
Glasgow et al.’s 5 A’s model of self-management support [11]

The 5 A’s model (Fig. 1) entails five steps, namely:

1. Assess: Assessing the patient’s knowledge, beliefs, and behaviors;
2. Advise: Advising the patient by providing specific information about the disease and information about the patient’s health status in an understandable manner so the patient can relate their self-management skills and behaviors to their health status;
3. Agree: Agreeing on goals collaboratively set with the patient and according to the patient’s priorities;
4. Assist: Assisting the patient by identifying and resolving barriers that hinder the patient in achieving the set goals;
5. Arrange: Arranging follow-up via e.g. e-mail or telephone.

The model assists healthcare professionals in structuring self-management support within a dynamic and tailored process. The 5 A’s model was originally introduced for self-management support for patients. However, we believe the 5 A’s model to be relevant for informal caregivers as well.

For self-management support to be effective, it is important that it is provided by suitable healthcare professionals. Nurses in particular are appropriate providers of self-management support since empowering patients and enabling them to understand and cope with their disease or disability, its treatment, and its consequences are core competences for nurses [14, 15].

Nurses are indeed main providers of self-management support in practice in the Netherlands and other European countries [16]. Some previous studies focused on nurses’ self-management support for patients with cancer or a variety of chronic conditions, and/or for their informal caregivers (e.g. Johnston et al. [17], Hammer et al. [18], Kaltenbaugh et al. [19], Northouse et al. [20], Verkaik et al. [21], Been-Dahmen et al. [22]). However, these studies often describe effects of interventions rather than nurses’ experiences and perspectives on self-management support to people in the advanced stage of an illness, or more specific, in the advanced stage of cancer, which is our main focus. Research on self-management support in people with advanced and ultimately fatal illnesses, apparently is still a rather young research area [23]. To our knowledge, no research has been done on how nurses experience and perceive self-management support specifically to patients and informal caregivers facing advanced cancer. Also the role of eHealth appears unexplored within this specific scope and target group.

For self-management support to be effective, it is essential that it is tailored to the recipient’s needs and properly facilitated [24]. In this regard, eHealth in the form of web-based or smartphone applications might be useful, supplementing or (partially) substituting for face-to-face self-management support by professionals. Furthermore, eHealth could be of particular added value for people with reduced mobility and who are too ill to travel [25]. Several studies have already investigated the effects of eHealth for cancer patients and informal caregivers, and their attitudes to eHealth along with the attitudes of various healthcare professionals (e.g. Slev et al. [26], Lubberding et al. [27], Paul et al. [28]). Some studies specifically investigated eHealth for people with life-limiting illnesses (e.g. Johnston et al. [25], Neergaard et al. [29], Collier et al. [30]). However, there appears to be a lack of studies focusing on nurses’ opinions about the use of eHealth specifically for self-management support for people confronted with advanced cancer.

We performed an online focus group study to gain more insight into how nurses perceive their role in self-management support for people confronted with advanced cancer and their opinions about the use of eHealth in this regard. The following research questions are addressed in this paper:

1a) How do nurses in the Netherlands currently support patients and informal caregivers facing advanced cancer in their self-management of problems and symptoms related to advanced cancer (e.g. pain, fatigue, loss of appetite, sadness/depressive moods, and anxiety, and a high care burden)?

1b) How would these nurses support the self-management activities of patients and informal caregivers in the ideal situation? Are there any discrepancies between the current situation and the ideal situation?

2) What are experiences and expectations of these nurses regarding the use of eHealth for self-management by or self-management support for patients and informal caregivers facing advanced cancer?

## Methods

### Recruitment and sample

In the Netherlands, three categories of nurses can be distinguished according to their level of education: registered nurses (RN) with secondary vocational education; registered nurses with higher professional education (Bachelor’s degree); and ‘nurse specialists’ with a Master’s degree in Advanced Nursing Practice. All three categories of nurses can follow specific continuing education courses in, for instance, oncology nursing or palliative care. Hereafter the term ‘nurse’ will be used interchangeably for registered nurses with secondary vocational education or higher professional education (Bachelor’s degree) and nurse specialists with a Master’s degree, unless otherwise specified.

Nurses were eligible for inclusion in the study if they 1) work with patients with advanced cancer on a daily basis, and 2) work in a hospital, home care, transmural care or hospice setting.

Nurses were recruited via open calls placed on social media (Facebook, Twitter) and via e-mails directly sent to nurses (*N* = 45) in the authors’ own professional network (LinkedIn). Additionally, announcements were placed on the website and the social media account of the Dutch Oncology Nursing Society.

The recruitment resulted in 56 nurses showing their interest in participating in the study. Recruitment via LinkedIn appeared to be most successful as it yielded 36 positive replies. All potentially interested nurses (*N* = 56) were sent an information letter by e-mail explaining the study objectives and methods. Ultimately, 11 of these 56 nurses did not participate due to e.g. personal circumstances or not having enough working experience with people with advanced cancer, or because they did not post any comments to the questions posed in the online focus group. These nurses were considered as non-participants. This resulted in a sample of 45 nurses working in different parts of the Netherlands, and in rural as well as urban areas. None of the participating nurses were close private or professional contacts of the authors. The participants were divided into six online focus groups. Table 1 depicts the participants’ characteristics.

**Table 1:** Characteristics of the participating nurses (*N* = 45)

Sex	
Male	1
Female	44
Mean age (range) (*N* = 36)	45.3 (25-62)
Care setting	
Hospital	19
Home care	11
Hospice	9
Other (e.g. transmural)	6
Mean work experience as nurse in years (range) (*N* = 35)	22.4 (3-41)
Highest degree in nursing	
Master’s degree in Advanced Nursing Practice	15
Higher professional education (Bachelor’s degree)	23
Secondary vocational education	7
Additional education course	
Oncology and/or palliative care	23
No additional course in oncology or palliative care	12
Unknown	10

### Data collection

Data were collected through online focus group sessions. Online focus groups are a relatively new online method for qualitative research using a group of participants [31]. An asynchronous form of the online focus group was used, meaning that participants could log into a secured website and respond to questions of the executive researcher (VNS), and posts of other participants, at times of their choosing, 24 h a day [32]. All online focus groups lasted 2 weeks.

Anonymity and protection of participants’ privacy were ensured by using aliases and personal login names and passwords.

Six, of which four ran simultaneously, asynchronous online focus groups were organized: one for registered nurses working in a hospital, one for registered nurses working in a home care setting, one for nurse specialists working in a hospital, one for registered nurses working in a hospice and two mixed groups of registered nurses and nurse specialists working in a hospital, home and/or hospice setting. The study started with the first four homogeneous online focus groups. The two heterogeneous online focus groups started 1 week later, while the first four were still running.

The decision was made to have both homogeneous and heterogeneous online focus groups in order to provoke discussion and potentially elicit different opinions regarding self-management support and eHealth for advanced cancer patients and their informal caregivers.

Semi-structured open ended questions concerning self-management support in dealing with physical and psychological complaints, and decision making regarding care and treatment were placed in discussion threads on the secured websites of the online focus groups. Some examples of the questions are presented in Table 2.

**Table 2:** Examples of questions posted on the secured websites of the online focus groups

Physical symptoms such as fatigue, pain and loss of appetite are common in advanced cancer cases. These symptoms can have consequences for people with cancer and their informal caregivers.
• Do you recognize this description?/Is this description familiar?
• What do you do at present to support these patients and/or informal caregivers in dealing with these physical symptoms (=aspects of self-management support)? And how would you want to do this in the ideal situation?
• What are your thoughts on the use of eHealth in this context?
Advanced cancer can be associated with somber moods, anxiety and uncertainty. Both the person with cancer and their informal caregivers may have these feelings.
• Do you recognize this description?/Is this description familiar?
• What do you presently do to help these patients and/or informal caregivers deal with these feelings (=aspects of self-management support)? And how would you want to do this in the ideal situation?
• What advice do you give patients and/or informal caregivers for situations where they would like to talk to a healthcare professional or caregiver but where this is not possible or only to a limited extent?
• What are your thoughts on the use of eHealth in this context?

The posed questions were directly related to the main research questions (see ‘Background’), and were based on e.g. the existing literature on the most prevalent symptoms in patients with advanced cancer [2]. The questions were drawn up in consultation with all members of the project group which encompasses, amongst others, four professionals with a nursing background (VNS, CME, ALF and HRWP).

Like in traditional face-to-face focus groups, not everyone had to respond to every question. Nevertheless, on the first page of each online focus group where instructions for participation were set out, and in e-mail alerts which were sent after a new question was posed, participants were asked to login regularly and were stimulated to respond to the presented questions, to comment on other participants’ posts, and to look back and respond to previously posed questions.

### Data analyses

A qualitative analysis method was used that was inspired by thematic analysis [33, 34]. Data analysis of the transcripts commenced as soon as the first online focus groups started, as part of a cyclical process of collecting data, analyzing data, collecting new data and so on.

Every day and multiple times a day, the executive researcher (VNS) logged in into the websites of the online focus groups, to analyze the responses to the questions in the discussion threads. Where appropriate, questions were amended or added to, depending on the responses of participants in the online focus groups. Hence, data collection and data analysis commenced as soon as the first participant responded to the first question placed in the first discussion thread.

First, all transcripts were read and re-read. The full transcripts of the discussions were automatically generated from the websites of the online focus groups, and concerned the literal responses of the participants. Next, open inductive coding was performed in order to identify important themes and subthemes related to the research questions. These themes and subthemes were then deductively categorized in accordance with the 5 A’s model for self-management support (see ‘Background’). Negative data, i.e. data that did not fit the five A’s, were not found. All themes could be ordered using the model. However, it was not always possible to fit themes exclusively in one specific ‘A’ as it applied to several A’s of the 5 A’s model.

The executive researcher (VNS) analyzed all the transcripts for the six online focus groups. To deepen and validate her analyses, two co-authors (HRWP and ALF) each independently analyzed one half of the transcripts. The main themes and subthemes were then finalized through discussion [33]. The interim and final analyses were also discussed with the other authors, who all read at least one transcript.

## Results

### Number of posts

On average, 41 posts were placed in each online focus group. The number of posts per participant varied from 1 post to 12 posts (on average 6 posts per participant).

### Self-management support by nurses

In this section, the themes resulting from the analyses of the transcripts are presented and categorized in the ‘Assess’, ‘Advise’, ‘Agree’, ‘Assist,’ and ‘Arrange’ steps in the 5 A’s model [11] (Table 3). Table 3 also shows the extent to which the current situation matches the ideal situation, as outlined by the participants in the online focus groups.

**Table 3:** Current situation and ideal situation regarding nurses’ self-management support in the case of advanced cancer

5 A’s	Current situation		Ideal situation	
	*Self-management support for patients*	*Self-management support for informal caregivers*	*Self-management support for patients*	*Self-management support for informal caregivers*
Assess	Obtaining an understanding of the patient’s background, personal situation, wishes, and needs by initiating a discussion, enabling an open discussion about topics, actively asking follow-up questions, listening	-^a^	More time	In general: More attention
Advise	Giving information and guidance, advising, listening, and referring the person to other disciplines or organizations	Giving information and guidance, advising, listening	-^a^	
Agree	Jointly setting goals, letting patients prioritize symptoms themselves	-^a^	-^a^	
Assist	Mapping barriers and strategies applied in the past, giving practical tips	-^a^	-^a^	
Arrange	Continuity of care	-^a^	In general: Better cooperation between intramural and extramural healthcare	
Throughout all 5 A’s			More attention to self-management support in the home situation	

^a^ no information from online focus groups that relates to the ‘A’ in question

#### *Assess*

Nurses in all the care settings covered said that when talking to patients with advanced cancer, they always try first to gain an understanding of their background, personal situation, wishes, and needs. Nurses find this important because they can only offer the desired, optimal self-management support if they are aware of the patient’s background and issues.

Nurses also said that obtaining a picture of the patient’s situation makes things clearer for the patient too, and this helps generate or enhance self-awareness.

*“When we ask for information, patients find that they reflect on things more.” (nurse specialist)*

Nurses said that the patient’s insight into their own situation and functioning puts the patient more in control of what is happening. This lets the patient take charge and/or stay in charge, which helps in tackling the issues at stake. Nurses also mentioned that if the patient and the informal caregiver have a good picture of the situation, this creates mutual understanding between them. Mutual understanding can improve the communication between the patient and the informal caregiver; any misunderstandings and confusion can be straightened out. This also often improves communication between the patient, the informal caregiver, and the nursing professional.

Nurses assess the patient’s issues and support needs by initiating a discussion, making it possible to talk openly about topics, proactively asking follow-up questions, and listening.

*“What I do now is first ask what the problem is, how important is it for the patient, get to know the patient well so that I can give advice that suits their situation.” (nurse specialist)*

Some nursing professionals said that they use screening tools when assessing the patient’s symptoms, for example the Utrecht Symptom Diary which is a Dutch translation of the Edmonton Symptom Assessment System [35], and the Lastmeter, the Dutch version of the Distress Thermometer [36].

*“What we do, is we let the patient fill out a Utrecht Symptom Diary, so the patient gets insight in the symptoms he suffers from.” (hospital nurse)*

*“In practice, the Distress Thermometer is being used which also gives insight in not immediately discussed feelings.” (nurse specialist, in the context of support with dealing with psychological problems)*

The screening results can present angles from which to start discussing issues. However, others also emphasized that running through the screening tools should never be an end in itself, that nurses must not blindly trust the figures.

*“I am also somewhat anxious about translating complaints or symptoms into scores or numbers. It could be used as a starting point […] but not more than this.” (nurse specialist)*

The discussion techniques that nurses use to obtain a picture of the patient’s background depend on the nature of the issues—physical, psychological, or spiritual/existential. In the case of physical problems, nurses said that actively asking follow-up questions is often the best way to determine the nature and cause of physical symptoms. This is in part because some of these symptoms may be due to psychological or social problems, such as increasing pain caused by too many family visits.

Some also mentioned that it can be necessary to proactively initiate a discussion about anxiety, for example, or somber moods, uncertainty and distress about the prognosis. They say that patients do not always speak out about these feelings, even not to their informal caregivers.

*“Getting a conversation going (if people allow that) can make people feel relieved and sometimes they learn how to understand each other’s emotions better.” (transmural nurse)*

According to the nurses, whether the symptoms listed above are eventually discussed in detail depends on the patient’s needs.

Nurses added that the actual situation in this regard is close to what they would ideally do. Even so, they stressed that they would like more time—with the exception of a number of home care nurses whose organizations offer the option of ‘continuity visits’ (home visits following discharge from hospital). Nurses emphasized that with more time, they could assess the patient’s interests, needs, wishes, and cognitive capacity better, as well as the disease stage, in order to improve tailoring of self-management support:

*“The ideal situation would be that I would be able to find out what skills the patient has that are necessary for self-management and work with the patient and/or informal caregiver to determine interventions that tie in with that.” (nurse specialist)*

Furthermore, nurses in hospices in particular said that in the ideal situation more attention would be given to assessing the informal caregivers’ situation:

*“In the ideal situation, we hospice staff would be better informed about the informal caregivers’ hobbies, social activities and how they deal with social contacts [...] The combination of this [ed. combination of care for a patient and continuing with their ‘own’ social activities] and ensuring contact with their sick relative in the hospice is so important for the informal caregivers in particular.” (hospice nurse)*

#### *Advise*

Nurses said that helping advanced cancer patients deal with problems and symptoms in their daily lives involves giving them information and guidance, advising them, listening to them, and referring them to other disciplines or organizations. As with ‘Assess,’ this too is important in engendering and enhancing self-awareness and mutual understanding between the patient, their informal caregivers, and nurses.

Giving information and guidance is relevant for example in dealing with loss of appetite in the palliative phase.

*“The loss of appetite often causes a lot of frustration with one another and distress. I try [...] to explain how the loss of appetite is part of the disease process. I find that this takes some of the pressure off and that the client and their informal caregivers start to understand each other better again.” (transmural nurse)*

Nurses said that they provide information repeatedly in different forms (verbally, on paper, and digitally). This gives patients the opportunity to read the information several times, which helps them retain the information. Despite this, nurses felt that providing information deserves more attention. This applies in particular to providing clear, unambiguous information, and clear communication about the prognosis.

Nurses working in hospices sometimes said that they “*[…] involve informal caregivers in the talks as well. They [ed. informal caregivers] are also given support in the form of information about the extent to which symptoms are a part of the final stage of life and how they can continue to give support and care.” (hospice nurse).*

Nurses mentioned occasionally that they give practical tips that advanced cancer patients and their informal caregivers can apply at home, mainly with regard to physical symptoms. For example, if a patient is low on energy, nurses advise the patient to draw up a schedule of activities and concentrate on allocating their energy well.

*“We explain about dividing energy and taking into account the, to the patient, important moments, e.g. visitors, hobbies, etc. In practice, it appears that the patient mentions having hobbies, but that hobbies get put on hold because of low energy. A daily schedule can help to save enough energy for this.” (hospice nurse)*

In the case of psychological symptoms, nurses often deliberately refrain from giving advice and offering solutions. They said that somber moods, anxiety, uncertainty, distress, and worry are feelings that cannot be alleviated and that each individual patient deals with this in their own way. Nurses concentrate mainly on listening, acknowledging these feelings, and being there for the patient. According to nurses, these are the best approaches for supporting patients with symptoms of this nature.

*“[...] leaving room for everything they are feeling, thinking and experiencing, not giving each other advice and not coming up with solutions. Anything is allowed.” (transmural nurse)*

Some do give a few tips to the patient, such as talking about the symptoms and looking for diversion.

Furthermore, nurses said that they refer patients to other disciplines, such as a psychologist or spiritual caregiver, to help them deal with psychological problems. Hospice nurses in particular also frequently mentioned pointing out the options for complementary care, such as creative therapy, aromatherapy and massages for both psychological symptoms and physical symptoms. They said that patients derive energy from this.

Nurses gave few examples—even after follow-up questions—of how informal caregivers are supported in dealing with the patient’s problems and symptoms or their own problems. Only some hospice nurses and home care nurses gave examples in this regard.

*“Informal caregivers’ feelings of powerlessness are often an issue here. They already have to hand over a lot of things when their relative is admitted to a hospice. [...] We often then look for alternative responsibilities for the relatives [...]. For instance, you can explain how to give good oral care. Complementary care, such as giving a hand massage, can also be handed over to relatives to some extent.” (hospice nurse)*

*“We support informal caregivers by listening and giving tips and advices. For example […] by taking the pressure off nutrition. My experience is that informal caregivers feel like they are not giving proper care, if the ill one eats insufficiently. We also offer voluntary palliative care so informal caregivers could unwind a little.” (home care nurse)*

#### *Agree*

According to the 5 A’s model, collaborative goal-setting is part of the ‘Agree’ step. However, nurses barely mentioned setting goals in partnership with an advanced cancer patient and/or informal caregiver, or the use of an individual plan. Nurses did mention that wherever possible they look at what the patient’s wishes are and that they let the patient decide which symptoms and/or feelings or problems should be given priority. Nurses emphasized that it is important to do this with the patient because this lets the patient remain in control as much as possible, or puts the patient in control if that was not already the case.

*“When getting insight in the patient’s problems, it is also important to know what is important to the patient himself, to work on. What does the patient experience as the biggest issues.” (nurse specialist)*

For example, when decisions have to be taken, nurses support the patient by helping them to draw up a list of advantages and disadvantages and weigh these up against one another, and to write down any questions for the next appointment with the treating physician, family doctor or nurse.

*“Patients sometimes ask then what they should do. I can’t give them that advice but I can help them to get an overview of everything. It helps enormously if they write this down on paper and e.g. assign a degree of importance.” (hospice nurse)*

#### *Assist*

According to the 5 A’s model, an important aspect of this step is assisting in developing plans to meet goals. This also implies mapping any barriers that might prevent the patient or informal caregiver from achieving the goals, deploying interventions and giving practical advice that can help them achieve the defined goals. A number of nurses mentioned that when dealing with patients with advanced cancer, they assess whether there are barriers, for example in dealing with feelings of anxiety, somber moods, and uncertainty, and if so, what strategies the patient has for removing those barriers.

*“[...] how did you respond to difficult situations in the past and what helped you then to get back on track?” (home care nurse)*

Nurses stated that every patient is unique and deals with their feelings, symptoms and problems in their own way; that is one reason why it is important to put the patient in control when dealing with symptoms. Patients often know best themselves where their strengths lie. If that is not the case, the patient will need assistance, to be made more aware of their own strengths by becoming actively involved in their own care.

#### *Arrange*

Nurses did not explicitly state how they arrange follow-up. The only point made by some is that they sometimes encourage patients to write down goals and questions so that these can be referred back to in a subsequent consultation.

Nurses stressed the importance of follow-up primarily in terms of the continuity of care. In the ideal situation it would not just be about the continuity of the care they are delivering; their care would be part of a multidisciplinary collaborative approach within and between intramural and extramural care providers. This would ensure follow-up in the home situation as well.

*“Home visits should also be much more effective. This currently depends on the hospital and partnerships with home care organizations. The hospital can also inform the primary care side and make sure the family doctor is aware of the bad news at an earlier stage and that the oncological or palliative care nurse makes contact. So that needs better cooperation between the primary care and the hospital.” (home care nurse)*

#### *Throughout all 5 A’s*

Regarding self-management support in the ideal situation, hospital nurses said that self-management support should be extended to include dealing with problems when at home.

*“I think one point for improvement would be instructing people in the hospital where they can find information/support themselves to make it easier for them to tackle this when they get home. There should be more continuity here; at the moment the hospital and the home are two separate worlds. [...] More continuity too in information and so on; there are loads of different information sources at the moment and patients can no longer see the wood for the trees.” (hospital nurse)*

### Experiences with and opinions on the use of eHealth in self-management support

Nurses said they do not often use eHealth. They do see potential added value from eHealth, both for general healthcare information and for disease-specific information and practical advice. Some mentioned that it is important that patients can choose their own topics, that the eHealth application has an appropriate design for the target group, that it is available on smartphones, computers, and tablets, and that there are options for printing.

Nurses also said that eHealth can let patients remain in control, for example if there is a digital symptoms diary or the ability to view your own health record, if it makes it easier to ask a healthcare professional questions, or if it enables online contact with peers.

*“Use of a symptoms diary can certainly be worthwhile and could be part of an eHealth program. Using this can also give a patient a better understanding of their symptoms, and they may be able to make their own connections between activities and symptoms.” (hospital nurse)*

*“[...] precisely for those who want to remain self-reliant for as long as possible. A digital patient record with the patient as the owner could be particularly beneficial in letting the patient be in control.” (hospice nurse)*

Hospital nurses in particular said that eHealth could promote and safeguard the continuity of care if there is a link between the eHealth application and the physician, family doctor, and/or nurses. Moreover this would ensure the accuracy and clarity of the information.

However, nurses also made qualifying remarks. For instance, they repeatedly emphasized that eHealth cannot and should not replace personal contact. They therefore prefer a combination of eHealth and personal contact with a healthcare professional.

Nurses also said that eHealth is not suitable for everybody. Some hospice nurses saw the main potential added value of eHealth in the care of patients in the early palliative phase. Patients often no longer have enough energy to use a laptop or tablet, for example, in the final phase. According to the hospice nurses, eHealth could still have added value for informal caregivers in the terminal phase.

*“However, I frequently see patients bringing their tablets, setting up a laptop but subsequently hardly having time/energy for it. Relatives possibly might benefit from it more.” (hospice nurse)*

Home care nurses said that eHealth is less suitable for the current generation of older patients because they do not know how to use computers and cell phones.

*“I frequently deal with (frail) elderly people (+75 years), 99% don’t have knowledge of controlling a PC, app or tablet. This would probably be different in the next generation of older people.” (home care nurse)*

Furthermore, some nurses said that eHealth is more suitable for support in dealing with physical problems than psychological problems. According to nurses, eHealth cannot remove or resolve feelings of somberness, anxiety and uncertainty, although putting tips online on how to deal with this could be worthwhile.

*“Of course a program with tips and tricks and elements to cheer people up would be OK. I don’t think anything fundamental can be done about somber moods, anxiety and uncertainty.” (hospital nurse)*

## Discussion

Within the scope of self-management support, Dutch nurses pay considerable attention, to the assessment of a patient’s background, personal situation, wishes, and needs (‘Assess’ in the 5 A’s model), and to the provision of illness-related information and advice (‘Advise’ in the 5 A’s model). This result is in line with the findings in the study of nurses working with patients with various chronic conditions by Been-Dahmen et al. [22].

Our study, however, also shows that nurses are not inclined to give advice about psychological problems; they tend mainly to listen to the patient and refer them to a psychologist or spiritual caregiver. This also fits with the findings of Been-Dahmen et al. [22], as well as with the systematic review by Ventura et al. [37] of patients receiving palliative care at home and their informal caregivers. That study concluded that nurses and other professionals provide better-targeted support for physical problems than for psychological problems [37].

The findings above are striking as paying attention to psychological problems is actually seen as an essential element of palliative care [38].

It is interesting to note that ‘Agree’ (collaborative goal-setting) and ‘Assist’ (assisting patients in achieving their goals) are barely mentioned by the nurses in our study, whereas these are essential aspects of self-management support. Nurses also seem to pay relatively little attention to follow-up as an aspect of self-management support (‘Arrange’ in the 5 A’s model). In a European study of how self-management support is integrated into the care for the chronically ill, Elissen et al. [16] also concluded that collaborative care planning and structured follow-up receive little consideration in practice. These are therefore areas for improvement.

Furthermore, it is noticeable that nurses currently pay little attention in their daily practice to self-management support for informal caregivers. This result is remarkable, given that support for relatives is an essential part of the care of the incurably ill (see the WHO definition) [38] and of self-management support (see Wagner et al. [39]). The above result, however, is not a new finding.

Previous research on oncology and palliative care also pointed to the fact that informal caregivers still are an underserved population [37, 40–42]. Explanations for this finding regard: Informal caregiving often is a gradual process, and relatives are not really aware of the fact that they are becoming an informal caregiver. Realization often comes later in the disease trajectory [40]. Once the caregiver role is acknowledged, most informal caregivers find it hard to discuss their own support needs in the presence of the cared-for person [41]. To overcome these barriers, consultations for informal caregivers alone, have to be arranged [41].

Still, there appear to be differences between settings in this regard: Hospital nurses hardly mentioned self-management support to informal caregivers, while some hospice nurses and home care nurses did mention this. Paying consideration to self-management support to informal caregivers, therefore, seems to be more of a matter for the latter mentioned group of nurses. Signs of stress and physical and psychological symptoms in informal caregivers might be more likely to be picked up in the home care or hospice setting [40]. Hospital nurses often mainly see the patient and are busy with technical tasks during the patient’s visit to the hospital or outpatient clinic or during treatment. Nurses in home care and hospice care may have a better picture of what the informal caregiver could do to cope with the impact of their relative’s illness on their daily lives. Because of the qualitative nature of this study and therefore the small sample size, we should be cautious on reporting ‘differences’ between nurses. Therefore, above mentioned findings have to be interpreted with prudence.

Furthermore, this study shows that nurses see benefits from eHealth. However they stress that it should never replace personal contact and that its applicability depends on patients’ digital skills, the disease stage and the nature of the problems and symptoms. Other studies [25, 29, 30] among both doctors and nurses working in palliative care came to similar conclusions. The finding that eHealth can enhance the patient’s control over things, for example by letting the patient record and monitor their symptoms online, is also backed up by studies by Collier et al. [30] and Johnston et al. [25]. The nurses in our study do not see a role for eHealth in the self-management of psychosocial problems such as anxiety, uncertainty, and somber feelings. Such views did not emerge in the aforementioned studies and contradict the support on the effectiveness of web-based psychological interventions in diverse patient populations [43, 44].

This study indicates that nurses value self-management support. However, sometimes they appear to omit providing practical advice, and they seem to pay little attention to the A’s of ‘Agree’, ‘Assist’ and ‘Arrange’ of the 5 A’s model. The fact that the steps in the 5 A’s model were not explicitly mentioned in the questions in the online focus groups may have contributed to this outcome. Findings might have been different if we asked directly about the A’s of the 5 A’s model.

We intentionally chose to include practical descriptions of ‘self-management’ and ‘self-management support’ rather than definitions, to avoid differences in participants’ interpretation of self-management and self-management support. However, the data yielded may have been constrained by the nurses’ perception of self-management support. If self-management support in nurses’ understanding of the concept, does not include e.g. the provision of practical advice, collaborative goal-setting and arranging follow-up, then perhaps it is logical that these elements were not discussed. Despite, one could expect that at least some nurses would refer to the essence of the steps in the model as the 5 A’s model is a starting point in the Dutch national care standard on self-management, and because self-management support is mentioned as a core task of today’s nurses, in the national report on nursing roles in the Netherlands [15].

### Strengths and limitations of this study

For this study we used a combination of convenience and purposive sampling. To involve nurses with different backgrounds, we approached and eventually included nurses working in various care settings, in different areas of the Netherlands and with differences in years of working experience. We prevented that only nurses with a specific interest in self-management support participated, as we did not use ‘providing self-management support’ or ‘being acquainted with self-management support’ as inclusion criteria. None of the participating nurses were close private or professional contacts of the authors.

Another choice made in this study was to opt for online focus groups rather than traditional face-to-face focus groups. This choice was made, primarily for practical reasons: nurses are often very busy and prefer not to spend time traveling to a location for a traditional focus group. In general this worked well. We were able to recruit enough nurses to gain a picture of how nurses offer self-management support for dealing with the symptoms and problems that people may encounter when faced with an incurable form of cancer. Given that the final online focus group did not produce any significant new information, we can assume that we achieved data saturation.

In the course of the 2 weeks that each online focus group was active, we added further in-depth questions. Moreover, we sometimes repeated questions for debate and added a question about a specific example. Some participants did not log in for every new question and this could mean that some of the in-depth questions or repeat questions were not read by all the participants. This is a limitation of online focus groups when compared with traditional face-to-face focus groups.

## Conclusions

The nurses in this online focus-group study value self-management support and eHealth for advanced cancer patients. However, they seem to disregard important elements of self-management support, such as providing practical advice, collaborative goal-setting, and arranging follow-up. At present little consideration is given to self-management support for informal caregivers. We recommend making nurses more aware of the importance of self-management support for both patients and informal caregivers. This awareness could be achieved through targeted (re)training of nurses in self-management support and the 5 A’s model using the Dutch national care standard as starting point, and incorporating self-management, self-management support and the 5 A’s model as integral part of nursing education.
